# Revisiting the role of *Akkermansia muciniphila* as a therapeutic bacterium

**DOI:** 10.1080/19490976.2022.2078619

**Published:** 2022-05-25

**Authors:** Jiyeon Si, Hyena Kang, Hyun Ju You, GwangPyo Ko

**Affiliations:** a Institute of Health and Environment, Seoul National University, Seoul, Republic of Korea; b Department of Environmental Health Sciences, Graduate School of Public Health, Seoul National University, Seoul, Republic of Korea; c Center for Human and Environmental Microbiome, Institute of Health and Environment, Seoul National University, Seoul, Republic of Korea; d KoBioLabs, Inc, Seoul, Republic of Korea; e Bio, Seoul National UniversityBio-MAX/N-, Seoul, Republic of Korea

**Keywords:** *Akkermansia muciniphila*, molecular mechanisms, biotherapeutics, metabolic inflammation, effective compounds

## Abstract

Despite a short history since its first isolation, *Akkermansia muciniphila* has been extensively studied in relation to its effects on human metabolism. A recent human intervention study also demonstrated that the bacterium is safe to use for therapeutic purposes. The best-known effects of *A. muciniphila* in human health and disease relate to its ability to strengthen gut integrity, modulate insulin resistance, and protect the host from metabolic inflammation. A further molecular mechanism, induction of GLP-1 secretion through ICAM-2 receptor, was recently discovered with the identification of a new bacterial protein produced by *A. muciniphila*. However, other studies have suggested a detrimental role for *A. muciniphila* in specific host immune settings. Here, we evaluate the molecular, mechanistic effects of *A. muciniphila* in host health and suggest some of the missing links to be connected before the organism should be considered as a next-generation biotherapeutic agent.

## 1. Introduction

Advances in large-scale sequencing methods have enabled the investigation of whole microbial communities and has stimulated research on the human gut microbiome. In particular, the gut microbiome has been extensively investigated in relation to its association with clinical aspects of metabolic disorders.^1,2^ Obesity was one of the first host pathologies demonstrated to be clearly associated with particular gut microbial ecologies^3,4^ and was the metabolic disease for which *A. muciniphila* was first reported to exhibit a beneficial effect on host physiology.^5^

**Figure 1.**
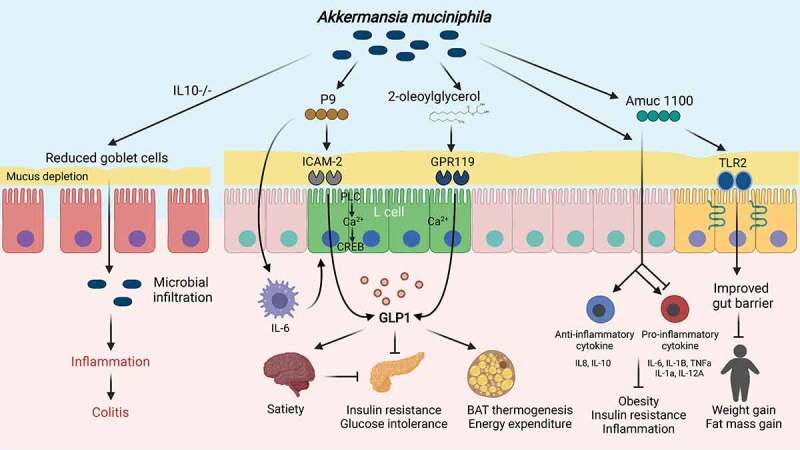
***Akkermansia* regulation of host metabolism**. An overview of the molecular mechanisms for its action in the gut. Activation of TLR2 by Amuc 1100, an outer membrane protein of *A. muciniphila*, improves the epithelial tight junction and decreases body weight and fat mass. The protein can also trigger a range of anti- and pro-inflammatory cytokines, preventing obesity, insulin resistance, and inflammation in the visceral adipose tissue. A hormone induced by L cells, glucagon like peptide-1 (GLP-1), is implicated in satiety and glucose homeostasis. *A. muciniphila* regulates GLP-1 by increasing an agonist (2-oleoylglycerol) of the GPR119 endocannabinoid receptor. P9 protein produced by *A. muciniphila* binds to intercellular adhesion molecule-2 (ICAM-2) and activates phospholipase C (PLC), intracellular Ca^2+^ signaling, and CREB. P9-stimulated IL-6 expression in macrophages is involved in GLP-1 production. Repeated oral gavage of *A. muciniphila* promotes inflammation in IL10-/- mice as a result of increased microbial infiltration due to reduced numbers of mucin-filled goblet cells. The figure was created using BioRender.com.

*Akkermansia* is a Gram-negative, oval-shaped, non-motile, and oxygen-tolerant anaerobic bacterium with only a short history of investigation due to its relatively recent isolation.^6,7^ Derrien *et al*. (2004) first isolated *Akkermansia muciniphila* (type strain Muc^T;^ ATCC BAA-835), a member of the *Verrucomicrobia* phylum in the gut of humans and animals.^6,8,9^ It was found to account for 1–3% of the total gut bacteria and persists throughout the life of the colonized host.^10,11^ *A. muciniphila* is mainly localized to the distal parts of the small and large intestines, where it utilizes mucin as its main energy source to provide the amino acids and sugar groups required for bacterial growth.^6,12–14^ It is known that the bacterium has the enzymatic capability to degrade the complex mucin structure.^15,16^ When *A. muciniphila* degrades mucin, it releases less complex carbohydrates from the mucin layer and produces organic acids such as acetate and propionate.^6^

During searches for bacteria beneficial to human health, *A. muciniphila* has emerged as one of the most promising biotherapeutic agents, since it has been shown to confer multiple beneficial effects in relation to certain metabolic diseases. These effects include the improvement of metabolic parameters, the strengthening of gut integrity, the stimulation of gut peptide hormone secretion, and the amelioration of metabolic inflammation.^5,17,18^ Such clinical benefits have resulted in the bacterium being tested for safety in human intervention studies.^19,20^ It was presumed to be safe after live or pasteurized *A. muciniphila* at 10^10^ cells were supplied daily for 3 months in overweight/obese individuals, without adverse effects.^19^ The safety of the bacterium was also confirmed in patients with type 2 diabetes (T2D).^20^ Here, we briefly review the current molecular mechanisms attributed to *A. muciniphila* and revisit its role in metabolic diseases. We also explore missing links to be filled before this organism could be considered as a biotherapeutic agent.

## 2. Molecular mechanistic function of *A. muciniphila* in metabolic diseases

A number of studies have provided evidence that *A. muciniphila* plays an important role in the regulation of host metabolic processes associated with metabolic disorders.^5,21–23^ In particular, the ability of *A. muciniphila* to reduce gut permeability has been identified as a major mechanism by which it regulates host metabolism.^5,24,25^ While gut mucus captures bacteria and prevents epithelial abrasion as a first line of host defense, the tight junction barrier of the gut inhibits the leakage of bacterial antigens from the lumen into epithelial tissues.^26,27^ Although *A. muciniphila* is renowned for mucin degradation, it is also able to increase the production of mucin by increasing both the number and density of goblet cells in high fat diet (HFD)-induced mice, thus restoring the thickness of the mucus layer and thereby strengthening the intestinal barrier.^5,15,18^ Amuc 1100, an outer membrane protein of *A. muciniphila*, was reported to decrease body weight and fat mass while improving the epithelial tight junction in HFD-induced obese mice.^28^ A number of molecular mechanisms by *A. muciniphilia* contributes to the amelioration of metabolic diseases have been identified^17,18,29^ and further examples are discussed below.

### 2-1. Communication through gut peptide hormone secretion

The role of gut microbiota in modulating the release of intestinal peptide hormones that affect appetite and energy homeostasis has been highlighted by a number of previous studies.^30–32^ L cells, which are intestinal enteroendocrine cells, play a crucial role in the gut-brain axis. They detect the presence of nutrients, microbiota, and metabolites via a G protein-coupled receptor (GPCR) and respond by secreting gut peptide hormones in association with elevated intracellular calcium (Ca^2+^) concentration, which signals to the brain to regulate satiety.^33–35^ Specifically, glucagon-like peptide-1 (GLP-1), a hormone induced by L cells, has been shown to suppress appetite.^36^ GLP-1 is also categorized as an incretin hormone due to its role in postprandial glucose clearance, by facilitating insulin secretion from pancreatic beta cells.^37^ In addition, GLP-1 stimulates brown adipose tissue (BAT) thermogenesis via activation of the AMPK pathway, thereby increasing energy expenditure.^38^ Not surprisingly, due to its proximity to L cells, the gut microbiota plays a crucial role in enteroendocrine cell differentiation and gut hormone secretion, particularly GLP-1.^39^ For example, *A. muciniphila* treatment increased the level of 2-oleoylglycerol, an endogenous GPR119 agonist, in the mice ileum (Figure 1).^5^ Additionally, it has recently been shown that *A. muciniphila* supports GLP-1 release via a newly discovered protein, P9^17^. The molecular basis of the action of this novel protein will be further discussed later in this review.

### 2-2. Anti-inflammatory effects mediated by A. muciniphila

Previous research has hinted at an immunological relationship between *A. muciniphila* and host metabolic inflammation (Figure 1).^18,29^ In mice, daily oral administration of *A. muciniphila* after 4 weeks on a HFD, reduced visceral adipose tissue inflammation by increasing the fraction of regulatory T cells (Tregs) within the total CD4 T cell population and significantly reduced *IL-6* and *IL-1β* expression in the visceral adipose tissue.^18^ Given that IL-6 and IL-1β secretion are known to promote adipose tissue inflammation, and are linked to obesity and insulin resistance, it was proposed that this anti-inflammatory activity of *A. muciniphila* could be the mechanism by which it ameliorates these metabolic disorders.^40,41^ Since Tregs are known to be key players in the regulation of adaptive immune responses associated with metabolic disorder-induced chronic inflammation,^42,43^ *A. muciniphila*-induced increases in Treg populations could be another of the organism’s mechanisms for modulating such inflammation. Dysregulation of the adaptive immune system was also associated with *A. muciniphila* colonization. The organism was found to be prevalent in immunodeficient Rag1^−/−^ mice, lacking T and B cells, but bone marrow transfer from Rag^+/+^ to Rag1^−/−^ mice reduced the levels of *A. muciniphila* in their guts.^29^ The crosstalk between immune system and *A. muciniphila* is also a representative characteristic of its anti-inflammatory effects in the intestinal mucosal barrier.^44–46^ The abundance of *A. muciniphila* has been found to be markedly reduced in patients with inflammatory bowel disease (IBD), including Crohn’s disease and ulcerative colitis (UC), implicating the organism in protection against inflammatory intestinal injury.^44,45^ The effect of *A. muciniphila* in systemic and intestinal anti-inflammation was demonstrated in an *in vivo* study using a dextran sulfate sodium (DSS)-induced colitis mouse model.^46^ Daily oral gavage of *A. muciniphila* for 14 days improved colitis by significantly downregulating pro-inflammatory cytokines such as TNF-α, IL-6, IL-1α, and IL-12A in mouse serum and colon tissue. In addition, the level of the immunomodulatory cytokine IL-10 was significantly enhanced by *A. muciniphila* administration. As effective compounds, both the *A. muciniphila* proteins, Amuc 1100 and P9, were also found to have immunomodulatory and immunometabolic functions. *A. muciniphila* and Amuc 1100 induced a range of anti- and pro-inflammatory cytokines (i.e., IL-8, IL-6, IL-1β, IL-10 and TNF-α).^47^ P9 was demonstrated to have IL-6 dependent anti-obesogenic effects.^17^ It is intriguing that these two studies found the elevated level of IL-6 by *A. muciniphila* unlike other HFD or inflammation models. IL-6 is a pleiotropic cytokine that can be involved in both pro- and anti-inflammation through trans- and classic signaling, respectively.^48,49^ Although it is not yet investigated if the reduction/enhancement of IL-6 observed with *Akkermasia* is linked to these signaling pathways, the cytokine has been implicated in metabolic processes such as hepatic inflammation and glucose homeostasis via BAT.^50,51^ In human subjects, it was also recently shown to delay gastric emptying and, consequently, postprandial insulin secretion.^52^ As a potent known GLP-1 stimulator,^53^ IL-6 induced by *A. muciniphila* may follow additional mechanisms than being a direct anti-/pro-inflammatory cytokine in the host metabolic homeostasis, which warrants further studies.

## 3. Potential therapeutic role of *A. muciniphila* in neurological disorders

In addition to its favorable effects in the gut, recent studies have provided a link between *A. muciniphila* and several neurological disorders such as autism spectrum disorder (ASD), psychological disorders associated with IBD, refractory epilepsy, Alzheimer’s disease (AD), and amyotrophic lateral sclerosis (ALS).^54–58^ According to a meta-analysis based on five clinical studies, children with ASD have a lower abundance of *Akkermansia* in their gut than control groups.^54^ Although relevant mechanistic studies have yet to be undertaken, it is possible that Akkermansia may ameliorate the increased gut permeability seen in ASD patients.^59^ *A. muciniphila* was associated with a therapeutic effect on depression associated with IBD when administered in a colitis mouse model under chronic restraint stress.^55^ The authors also discovered that the fecal samples of UC patients with depression had a lower abundance of *A. muciniphila* than those from control UC patients, demonstrating the link between the intestinal bacteria and psychological disorders via the gut-brain axis. Co-administration of *A. muciniphila* and *Parabacteroides merdae* to ketogenic diet-fed mice resulted in protective effects from seizure via increases in the ratio of hippocampal GABA to glutamate.^56^ More specifically, changes in the two bacteria, promoted by the ketogenic diet, reduced gamma-glutamyltranspeptidase activity and colonic and serum ketogenic gamma-glutamylated amino acids, changing the metabolism of brain GABA/glutamate. Daily administration of *A. muciniphila* in an AD mouse model (i.e., APP/PS1), for 6 months, not only improved metabolic disorders including glucose homeostasis and lipid metabolism, but also AD-related symptoms such as amyloid β-protein deposition in the brain and cognitive impairment.^57^ However, the interaction of the brain with serum metabolites derived from *A. muciniphila* was involved in ALS, a neurodegenerative disease marked by the death of motor neurons.^58^ Notably, *A. muciniphila* supplementation in *Sod1*-Tg mice improved motor function and brain atrophy through the accumulation of *A. muciniphila*-associated nicotinamide in the central nervous system. These results suggest that *A. muciniphila* has a broad mechanistic potential to exert influence on both its residential and remote sites, thus highlighting the need to be cautious prior to using the bacteria in clinical settings.

## 4. Controversial points relating to immunometabolic functions of *A. muciniphila*

Although dozens of beneficial effects of *A. muciniphila* on human and mouse health have been reported, a few studies have challenged this positive role by showing possible adverse effects of the bacterium. A pilot study of pediatric type 1 diabetes mellitus (T1DM) patients revealed that the genus *Akkermansia* was significantly more abundant in patients with poor glycemic control (HbA1c >7.5%).^60^ In a genetically susceptible T1D mouse model, it was shown that early-life antibiotic treatment accelerated T1D development and changed taxonomic composition of the gut microbiome including increases in the abundance of *Akkermansia*.^61,62^ This effect was seen even with a single course of pulsed antibiotic therapy, underscoring the significance of dysregulated immunological- and microbial gut environment in immunometabolic disorders.

Despite its known anti-inflammatory properties, several studies have claimed that *A. muciniphila* can promote colitis, depending on the immunological setting.^63–65^ Observations of enriched *A. muciniphila* on the colonic mucosa and in feces of mice treated with DSS questioned whether the organism plays a beneficial role in the gut.^64,65^ Another study demonstrated that repeated oral gavage of *A. muciniphila* led to the induction of colitis and intestinal inflammation in specific pathogen-free and germ-free IL10^−/−^ mice. By contrast, the non-mucin-degrading bacterium, *Bacteroides acidifaciens*, did not induce the same pathologies.^63^ When IL10^−/−^ mice were compared with wild-type mice, thinner mucus layers were observed in the IL10^−/−^ mice, leading the authors to hypothesize that the mucin-degrading capacity of *A. muciniphila* facilitates microbial infiltration to the intestinal mucosa and stimulates bacterial-driven inflammation (Figure 1). In their early study of IL-10-/- mice treated with *Enterococcus faecium*, Ganesh et al. reported an adverse role for *A. muciniphila*, since a decrease in *A. muciniphila* was associated with lower caecal inflammation.^66^ In a subsequent study, using a simplified human intestinal microbiota (SIHUMI) mouse model, it was demonstrated that the co-presence of *A. muciniphila* with *Salmonella enterica* Serovar Typhimurium (SIHUMI-AS) induced more severe inflammation than was seen in mice with *S*. Typhimurium only (SIHUMI-S).^67^ Reduced numbers of mucin-filled goblet cells and thinner mucus layers were found in SIHUMI-AS mice indicating that mucin degradation by *A. muciniphila* can exacerbate pathogen exposure in the host.

A beneficial role for *Akkermansia* is also disputed in multiple sclerosis and Parkinson’s disease, as patients with these diseases consistently exhibit higher levels of the bacterium than control groups.^68–72^ Exposure of peripheral blood mononuclear cells of healthy subjects to *A. muciniphila* induced Th1 lymphocytes differentiation, implying pro-inflammatory effects of the bacteria in the host.^69^ These findings emphasize the need to further elucidate the specific mechanisms and molecular targets through which *A. muciniphila* modulates host immunity in the gut environment where countless interactions between microbes exist. Thus, the effects of *A. muciniphila*, in a range of immunological settings should be extensively investigated before the organism is used for therapeutic purposes.

## 5. *A. muciniphila* as a biotherapeutic agent: the missing links for clinical translation

The beneficial effects of *A. muciniphila*, observed in numerous human cohort studies as well as animal models, led researchers to evaluate the organism in clinical trials. A translational clinical study that involved washed microbiota transplantation (WMT), an alternative method of fecal microbiota transplantation (FMT), demonstrated that 53.7% of Chinese IBD patients showed improvement in clinical parameters (e.g., stool frequency, rectal bleeding, and physician’s assessment) with significantly increased abundance of *A. muciniphila*.^73^ When the response group and non-response group were compared, the non-response group did not exhibit significant changes in the *A. muciniphila* levels, implying a prognostic role for this bacterium. A study group in Belgium carried out a human clinical trial to address the association between *A. muciniphila* and metabolic disorders.^19^ Although oral administration of *A. muciniphila* did not result in reductions in body weight, fat mass, or hip circumference, it did improve insulin sensitivity, reduce insulinemia, and reduce plasma cholesterol in overweight/obese insulin-resistant participants. Furthermore, pasteurized *A. muciniphila* showed slightly superior results than viable *A. muciniphila*. This human intervention study has significance as it paved the way for the organism to be used as a safe biotherapeutic agent. Another clinical intervention study in T2D patients in the United States also confirmed the safe use of a probiotic cocktail containing *A. muciniphila* for the improvement of postprandial glucose control.^20^ The findings of that study led to the development of market-ready *Akkermansia* products either as a sole bacterium or as part of a probiotic cocktail.

While most studies aiming to verify a biotherapeutic role for *A. muciniphila* have focused on its exogenous administration, they have overlooked the inherent abundance of the bacteria in the host. Indeed, it was recently reported that the abundance of *Akkermansia* varies with ethnicity.^73,74^ East Asians, for example, harbored lower levels of *Akkermansia* compared to Whites, although this was not necessarily linked to unfavorable metabolic features in a FMT experiment.^74^ With regard to the therapeutic administration of *Akkermansia*, it is important to establish whether endogenous *Akkermansia* abundance or the nature of *Akkermansia* strains is most critical for inducing metabolic changes in the host. To fully comprehend the role of *Akkermansia* in the prevention and treatment of immunometabolic diseases, below we suggest areas for further investigation.

### 5-1. Effective compounds in functionality of A. muciniphila

Since it was discovered that *A. muciniphila* was favorable to host health, researchers have investigated the bacterial components responsible for the benefits afforded by this organism. Extracellular vesicles (EVs), also known as outer membrane vesicles (OMVs), were the first *A. muciniphila* components to be evaluated.^75^ The protective effects of EVs, derived from *A. muciniphila*, were confirmed by multiple animal models and human cell lines.^24,75–78^ When a colon epithelial cell line (CT26) was pretreated with *A. muciniphila*-derived EVs (AmEVs), it reduced the pro-inflammatory cytokine production induced by *E. coli*-derived EVs.^75^ The authors additionally verified the anti-inflammatory role of AmEVs using a colitis mouse model. AmEVs also replicated the anti-obesogenic functions of *A. muciniphila* itself.^24,76^ In addition to beneficial effects linked to the gut, AmEVs were also reported to elicit potent anti-cancer activity via their immunomodulatory actions and effects on the gut-brain axis through serotonin production.^77,78^

Consistent effects on lipid disorders and insulin resistance, seen with pasteurized bacteria, led to the discovery of the *A. muciniphila* outer membrane protein, Amuc 1100.^28^ The mechanism of action of this protein was explained by its ability to activate TLR2 (Figure 1), in human peripheral blood mononuclear cells, and to increase the development of transepithelial electrical resistance in Caco-2 cells.^28,47^ Since the discovery of Amuc 1100, many favorable effects of *A. muciniphila* on host health have been attributed to the protein. These include anti-inflammatory and anti-tumorigenesis activity in DSS/azoxymethane treated mice,^79^ restoration of aberrant tryptophan levels in colitis mice,^80^ up-regulation of serotonin (i.e., 5-HT) metabolism in RIN-14B and Caco-2 cells,^81^ and improvement of depression-like behavior in chronic stress-induced mice.^82^ The beneficial health effects observed with a single, purified, *A. muciniphila* protein have expanded prospects for the commercialization of *A. muciniphila*, particularly in light of the difficulty encountered in culturing live, whole bacteria in bulk.

In an effort to find new, *A. muciniphila* targets with therapeutic potential, we recently identified the P9 protein and subsequently investigated its mechanism of action.^17^ We first confirmed its beneficial effects on body weight reduction and glucose tolerance and further revealed that it could promote thermogenesis in BAT and induce GLP-1 secretion. By contrast, Amuc 1100-induced GLP-1 secretion was not observed in this study. The mechanism of action of P9 related to its ability to bind to the intercellular adhesion molecule-2 (ICAM-2) and activate phospholipase C (PLC), intracellular Ca^2+^ signaling, and the cAMP response element-binding protein (CREB), rather than it acting as an agonist of the GLP receptor (Figure 1). This was the first demonstration of a link between ICAM-2 and GLP-1 secretion, as well as its activation by a bacterial protein. Additionally, it was observed that IL-6 was related to the GLP-1 secretion pathway induced by P9 treatment. P9 will need to be tested in additional disease models to see if it elicits the same effects via the same mechanisms, given that the protein has only been examined in a diet-induced obese mouse model. Further advances in our knowledge of the mechanisms by which P9 acts will help provide a fuller explanation of how *A. muciniphila* benefits host health.

### 5-2. Different functionality at the subspecies level

Several studies have used genome sequencing to investigate the genetic diversity of *A. muciniphila* isolates.^83–85^ In a study of human gastrointestinal tract metagenomic libraries, three out of 23 metagenomes were found to include distinct *Akkermansia* species, based on average nucleotide identity (ANI).^83^ The authors further used a validation twin cohort to suggest that different species of the genus *Akkermansia* can co-colonize the same host. Phylogenetic analysis based on 16S rRNA sequences and single nucleotide polymorphisms (SNPs) found in *A. muciniphila* genomes has identified a number of *A. muciniphila* clades.^84,86^ The three clades discovered, based on SNPs in different mammalian hosts (e.g., humans, mice, and pigs), were shown to have distinct functionalities such as phosphatidylinositol signaling system and amino sugar and nucleotide sugar metabolism. A recent genomic study based on metagenome assembled genomes (MAGs) discovered four clades of *A. muciniphila* (AmI, AmII, AmIII, and AmIV), with AmII synthesizing vitamin B12, which is required for succinate to propionate conversion.^87^ Another study investigated the phenotypes and functionalities of these clades, for example, growth rate, oxygen sensitivity, cell aggregation, and mucin fermentation.^88^ Interestingly, a large genomic analysis of *Akkermansia* (> 2,000 MAGs) identified five species-level clades, on the basis of their associations with different proportions of CRISPR spacers and matching bacteriophages, and revealed further information about the distinct functions of *Akkermansia* clades.^85^ The different functionalities exhibited by unique *A. muciniphila* clades, offers the opportunity to develop only those clades with the most desirable therapeutic benefits for clinical purposes. Analysis of MAGs reported sub-clades of *A. muciniphila* and clade-specific functioning without the need to cultivate these bacteria. However, it will be necessary to investigate whether or not there is an association between specific clades and beneficial effects in relation to immunometabolic diseases.

### 5-3. Crosstalk with other bacteria in the gut

On its own, *A. muciniphila* appears to be a potent host health modulator. However, in the complex gut environment, with trillions of residents, the organism is very likely to interact with other gut residents to elicit beneficial effects on the host. No changes in microbiota composition were observed in an overweight/obese human intervention study with live and pasteurized *A. muciniphila* treatments.^19^ However, factors known to affect the abundance of *A. muciniphila* in the gut, such as a HFD, fiber intake, medicine administration, antibiotics treatment, and immune dysregulation, all involve significant alterations in community diversity and composition. Moreover, several studies on the health effects of colonization by other probiotic bacteria, in both mice and human subjects, reported concomitant increases in the prevalence of *A. muciniphila*.^89–91^ In particular, Alard *et al*. found that a probiotic mixture containing *Lactobacillus rhamnosus* and *Bifidobacterium animalis* restored the levels of *A. muciniphila*, which was decreased in HFD-fed-mice while *L. rhamnosus* alone rather decreased the abundance of the organism.^89^ In various disease cases, an increase in *A. muciniphila* abundance was connected to the presence of other gut bacteria.^56,92–94^ Treatment of *C. difficile*-infected mice with *Bacteroides fragilis* resulted in concomitant increases in the levels of *A. muciniphila*, as well as higher mouse survival rates and increased gut barrier integrity.^92^ In IBD patients and allergic asthmatic children, the levels of both *A. muciniphila* and *Faecalibacterium prausnitzii* were shown to be lower than in non-disease control groups.^93,94^ Both of these organisms reside close to mucus layer in the intestine, and it has been suggested that oligosaccharides, vitamins, and acetate released from mucus, by *A. muciniphila*, support the growth of *F. prausnitzii*.^93^ Furthermore, co-administration of *A. muciniphila* and *Parabacteroides* in mice fed a ketogenic diet was shown to promote seizure protection, while the same protection was not observed with *A. muciniphila* alone.^56^ A co-culturing experiment demonstrated that sugars released by *A. muciniphila* indirectly increased butyrate producing bacteria (e.g., *Anaerostipes caccae, Eubacterium hallii*, and *F. prausnitzii*), and thus intestinal butyrate levels^95^ . Furthermore, the authors investigated the trophic interaction between *A. muciniphila* and *A. caccae* using a co-culture experiment followed by transcriptome analysis.^96^ In the presence of *A. caccae, A. muciniphila* upregulated its mucin-degrading genes. To date, only a few bacterial species have been experimentally examined for their cross-feeding effects with *A. muciniphila*. With increasing evidence of the ability of *A. muciniphila* to interact with a variety of other gut microbes, such co-culture experiments need to be expanded to include multiple species, to elucidate the extent of the metabolic interactions occurring in the gut that involve this organism.

## 6. Conclusions and outlook

Being one of the most abundant bacteria in the gut, along with the demonstration of its favorable effects on human health, *A. muciniphila* has sparked excitement as a promising biotherapeutic agent. However, an incomplete picture of the mechanisms by which it affects host metabolism, as well as contradictory data relating to its beneficial effects, cautions the need for further investigation before the bacterium can be considered as a safe, valuable and efficacious therapeutic agent for the treatment of immunometabolic disorders. The most well-studied effect of *A. muciniphila*, in the amelioration of metabolic diseases, is enhancement of the integrity of the intestinal barrier. More complex molecular mechanisms, such as GLP-1 induction, have only recently been discovered and one such mechanism is associated with the novel P9 protein produced by this bacterium. Until now, the significance of *A. muciniphila* in human health and disease had been limited to aspects of metabolic disease control. However, further investigation of the interactions of *A. muciniphila* with other gut bacteria may reveal metabolic crosstalk that impacts other diseases and host immunological conditions.

*A. muciniphila* has been extensively studied over a relatively short period of time. This has led to the discovery of its roles in metabolic disease amelioration, the identification of (as yet) incomplete mechanisms by which it achieves these beneficial effects, the evaluation of its efficacy in human intervention trials, and the identification of potential market/therapeutic products produced by the organism. With the safety of the organism proven in human subjects, the next objectives are to determine the appropriate treatment doses with whole bacterial cells or purified therapeutic components, and to confirm whether the molecular mechanisms of immunometabolism regulation observed in mice are also seen in humans. This is especially the case for those components not yet tested in humans, and for disease models where live/pasteurized bacteria did not achieve the desired external phenotypic modifications. With the promising outlook for *A. muciniphila*, as a therapeutic agent, we are set to identify the next generation of probiotics.
